# Characterizing Amino Acid Substitution with Complete Linkage of Sites on a Lineage

**DOI:** 10.1093/gbe/evab225

**Published:** 2021-09-28

**Authors:** Tristan L Stark, David A Liberles

**Affiliations:** Department of Biology and Center for Computational Genetics and Genomics, Temple University, Philadelphia, PA, USA

**Keywords:** positive directional selection, mutation-selection model, phylogenetic model, protein evolution

## Abstract

Amino acid substitution models are commonly used for phylogenetic inference, for ancestral sequence reconstruction, and for the inference of positive selection. All commonly used models explicitly assume that each site evolves independently, an assumption that is violated by both linkage and protein structural and functional constraints. We introduce two new models for amino acid substitution which incorporate linkage between sites, each based on the (population-genetic) Moran model. The first model is a generalized population process tracking arbitrarily many sites which undergo mutation, with individuals replaced according to their fitnesses. This model provides a reasonably complete framework for simulations but is numerically and analytically intractable. We also introduce a second model which includes several simplifying assumptions but for which some theoretical results can be derived. We analyze the simplified model to determine conditions where linkage is likely to have meaningful effects on sitewise substitution probabilities, as well as conditions under which the effects are likely to be negligible. These findings are an important step in the generation of tractable phylogenetic models that parameterize selective coefficients for amino acid substitution while accounting for linkage of sites leading to both hitchhiking and background selection.

**Significance**

The independent fixation of each site has been a strong assumption of phylogenetic models made for mathematical convenience. With this assumption, the effects of linked sites are ignored when estimating fitness profiles for amino acid substitution. This can lead to over or underestimation of selective effects associated with particular amino acid substitutions, as the effect of linked substitutions is averaged across the linked sites. Here, we take a first step towards accounting for the effect of linkage on amino acid fitness estimates by developing a model to treat linked sites and deriving results to detect circumstances in which linkage is likely to be present and need to be accounted for.

## Introduction

Computational prediction of protein-encoding genes that have changed functions under positive directional selection over the lineages of a phylogenetic tree remains one of the grand challenges in computational evolutionary genomics (Anisimova and Liberles 2012). Mutation–selection style models to perform this task have begun to replace d*N*/d*S*-based approaches for their greater mechanistic parameterization and insensitivity to d*S* saturation, but the development of improved codon models and mutation–selection models is ongoing (Spielman and Wilke 2015; Teufel et al. 2018). The mutation–selection framework creates a model for substitutions occurring along the branches of a phylogenetic tree by combining a model for mutation with a model for selection (Halpern and Bruno 1998). Since the original implementation, mechanistic mis-specifications of mutation–selection models have begun to be addressed, including parameterized mixture models of sitewise amino acid fitness profiles and relaxation of the equilibrium assumption (Teufel et al. 2018).

One of the important remaining mis-specifications is that of the nonindependence of sites. Nonindependence of sites because of structural and functional interaction can be described with chemical detail (see Grahnen et al. 2011). Nonindependence of sites due to linkage requires a population genetic framework that is distinct and is specifically addressed here. To enable future improvements to amino acid substitution models, including mutation–selection models, a better understanding of the dynamics of amino acid substitution with linked sites is needed. The effects of genetic linkage have been studied in population genetic models for some time. Early work by Griffing (1960), Nei (1963), Felsenstein (1965), and Hill and Robertson (1966) detailed the interactions between selection, linkage disequilibrium, and epistasis. In particular, an effect (widely known as the Hill–Robertson effect) by which linkage between sites leads to reduced efficacy of selection in finite populations was established by Hill and Robertson (1966). The Hill–Robertson effect can be conceptualized in terms of two parts, genetic hitchhiking (Smith and Haigh 1974) where less fit amino acids are fixed with higher probability due to more fit amino acids they are linked to and background selection (Charlesworth et al. 1993), where more fit amino acids are eliminated from the population without fixing due to less fit amino acids at other sites they are linked to. These linkage effects are distinct from nonindependence due to protein structural effects, which have previously received specific consideration in the context of amino acid substitution models (Parisi and Echave 2001; Robinson et al. 2003; Rodrigue et al. 2005, 2009; Dasmeh and Serohijos 2018). In contrast to protein-structural effects, the effects of linkage have largely been ignored in existing amino acid substitution models.

The parameterization of selective coefficients from mutation–selection models is informative about the nature of selective pressures. When implemented in a nonequilibrium framework (Blanquart and Lartillot 2008; Tamuri et al. 2009; Rodrigue et al. 2010; Rodrigue and Lartillot 2014; Jones et al. 2017; Parto and Lartillot 2017; Rodrigue and Lartillot 2017; Thiltgen et al. 2017; de Koning and De Sanctis 2018; Teufel et al. 2018; Kazmi and Rodrigue 2019; Ritchie et al. 2021), these models can be used to detect lineage- and site-specific selective pressures, including positive directional selection, dependent upon correct identification of selective coefficient parameter values. However, failure to account for the effects of genetic linkage could lead to misestimation of parameters, and hence to erroneous inferences of the strength of selective effects. It is an obvious conjecture that these processes could affect the parameterization of selective coefficients for sites which are treated independently, especially in regions of low recombination, leading to a dampening of the measured fitness differences corresponding to support for a neutral model when selective forces are operating.

The overall aim of this work is to understand the population dynamics associated with genetic linkage for ultimate use in the estimation of selective coefficients. We begin by introducing a finite-sites model, which, theoretically, is an appropriate substitution model for nonrecombining regions of the genome. Mutation rates can be inferred from a nucleotide-level model of mutation, and fitnesses could in principle be estimated from phylogenetic data in much the same way as in independent-sites mutation–selection models. However, although suitable for simulation of small population size data, the model is ultimately intractable, and hence, is not suitable for inference in practice. Therefore, we seek to develop a framework which captures the qualitative behaviors of this model in a more practicable way.

The main contribution of this paper is in the analysis of a simplified, infinite-sites model which is suitable for evaluating the extent of misestimation caused by fitting a site-independent mutation-selection model in the presence of linkage. We address the problem of evaluating the probability that several mutations *fix together* given that they do indeed fix over some branch of length *t_b_*. By fix together, we mean that the two (or more) mutations arise on the same lineage and that the haplotype introduced by the second (last) mutation then goes to fixation. This is done towards the goal of identifying which sites on which lineages should be treated as linked for the purposes of inferring fitness profiles. The natural link between population-level models that predict fixation and phylogenetic methods is the understanding that each branch reflects the evolution of a population over a period of time, the branch length.

## Results

### Finite Sites Model

Before we introduce the infinite-sites model which is of principle interest in this article, we first introduce a finite-sites equivalent. The finite sites model is fairly intractable due to its large state space; however, it would be suitable for simulations. Here, it is introduced to motivate the infinite-sites model upon which this article focuses. The main distinction between these models is if each mutation occurs at a distinct site or has some probability of occurring at a site that has already experienced a mutation.

Let X(t) be a Continuous-time Markov chain with *N *×* M* dimensional state space S′ tracking the evolution of a population of size *N* with genomes consisting of *M* sites; the state of the process is a matrix $X=[x_{ij}]$ with *x_ij_* tracking the allele in site *j* of individual *i*. We assume an alphabet A consisting of 20 characters, corresponding to amino acids. The order of the state space of the model is then $20^{MN}$, which is impractically large for even moderately large *M*, *N*. The model is nonetheless of some theoretical interest, and perfectly suitable for simulation.

For a state X∈S′ we denote the $k^{\text{th}}$ row of X, tracking the genome of the $k^{\text{th}}$ individual by *X_k_*. We write the (Darwinian) fitness of amino acid *A* at site *j* as $f_{j}(A)$, and we assume that fitnesses are multiplicative, so that the fitness of the $k^{\text{th}}$ individual is $f(X_{k})=\prod_{j=1}^{M}f_{j}(X_{kj})$. Furthermore, we let μ(A,B) denote the (site-independent) mutation rate from amino acid *A* to amino acid *B*. This process treats the continuous stepwise probability of losing an individual in proportion to the frequency of its genotype in the population and replacing that individual with the birth of a new individual in proportion to the cumulative fitness of individuals with that genotype in the population.

The process is characterized by the same dynamics as the Moran process (Moran 1958) with mutation, but for a fixed finite number of linked sites. More precisely, the generator of the process is given by $T=[t_{XY}]$, where the off diagonal elements are given by

(1) $$t_{XY}=\sum_{i,j}\frac{f(X_{j})}{N\sum_{k}f(X_{k})}R_{ij}(X,Y)+\sum_{i,j,A,B}\mu (A,B)E_{ij}^{AB}(X,Y),$$

where,

(2) $$R_{ij}(X,Y)=\{\begin{array}{cc} 1 & \;\text{if}\;X_{k}=Y_{k}\;\text{for}\;\text{all}\;k\neq i,\;\text{and}\;Y_{i}=X_{j}\\ 0 & \;\text{otherwise}\;, \end{array}$$

and

(3) $$E_{ij}^{AB}(X,Y)=\{\begin{array}{cc} 1 & \;\text{if}\;X_{kl}=Y_{kl}\;\text{for}\;\text{all}\;(k,l)\neq (i,j),\;\text{and}\;Y_{ij}=B,\;\text{and}\;X_{ij}=A\\ 0 & \;\text{otherwise}\;. \end{array}$$

The diagonal elements of T are as required to ensure zero row-sums.

That is, $R_{ij}(X,Y)$ is an indicator function which is 1 if and only if X and Y denote populations which are identical (in terms of the genomes of the individuals) with the exception of the $i^{\text{th}}$ individual—the $i^{\text{th}}$ individual of Y having a genome matching the $j^{\text{th}}$ individual of X. Similarly, $E_{ij}^{AB}(X,Y)$ is an indicator function which is 1 if and only if X and Y denote populations which are identical with the exception of the $j^{\text{th}}$ site in the $i^{\text{th}}$ individual, where character *A* is replaced with character *B* in Y relative to X. Equation 1 establishes the modeling framework, describing the transition through instantaneous death of individual *i* (*Y_i_*) and replacement with the birth of individual *X_j_*, in a population where all other individuals are unchanged, with the independent additive process of mutation of individuals. Equation 2 defines an indicator function to determine which states of the population can be reached from a given state *X* through the birth and death of individuals (i.e., only one individual can be replaced at a time, so the new state *Y* has to be the same in all but one individual). Similarly, equation 3 similarly defines the population states that can be reached through a mutation.

We now detail the infinite-sites model, which can be thought of as an approximation to the finite-sites model introduced above.

### Infinite-Sites Model

The infinite-sites model introduced here can be thought of as a combined model which transitions probabilistically between several simpler models, each tracking the evolution of the population with a fixed number of haplotypes. Initially, the model behaves as a Poisson process waiting for the first mutation to arise in the population. When the first mutation arises, the model then behaves as a two-allele continuous-time Moran model until the second mutation arises. There are, in general, four possibilities to be considered when the second mutation arises

- The first mutation may have gone extinct in the population—in this case, the process again behaves as a two-allele model.
- The first mutation may have gone to fixation in the population—this again leads to a two-allele model, wherein the “wild-type” now features the first mutation.
- The first mutation may still be segregating, in which case the process now behaves as a three-allele Moran model. This case further divides into two subcases:
- the new mutation may occur in the same lineage as the first, in which case we treat the fitness effects as multiplicative so that the three alleles have fitnesses $1,f_{1},f_{1}f_{2}$.
- the new mutation may occur in a wild-type individual, in which cases the three alleles will have fitnesses $1,f_{1},f_{2}$.

We reduce the number of possibilities which need to be explicitly modeled by conditioning on the number of substitutions which occur over the time period of interest and tracking only those mutations which lead to substitutions. Thus, we need only calculate probabilities associated with the event that the first substitution occurs before the second mutation arises, or that the first mutation is still segregating when the second mutation arises, and the second mutation occurs in the same lineage as the first. For example, applying the model over a rooted phylogenetic tree, for each branch we would track only those mutations which lead to substitutions on that branch, and we would ignore the effects of any haplotypes segregating on the branch besides the ones that lead to substitutions within the branch. The identity and number of substitutions occurring on a branch can be inferred through phylogenetic substitutional mapping procedures (see e.g., Monit and Goldstein 2018). In such a procedure, this would occur with a simpler model, but in principle could be performed with any model including the one from this paper for consistency.

The number of possibilities to be considered in the absence of conditioning is combinatorially increasing with the number of mutations which arise. However, conditioning on *K* substitutions occurring on a branch, and tracking only those mutations which eventually fix, the number of possible fixation histories is given by the number of compositions of *K*, $2^{K-1}$. We give the details for the case of three substitutions occurring on a branch below, which can be easily generalized to *K* substitutions.

For three substitutions, the possible fixation histories are: each mutation reaching fixation independently, the first two fixing together, the second two fixing together, and all three fixing together. Each history is associated with a different set of three submodels

- If the three mutations each fix separately, then we consecutively apply three two-allele models.
- If the first mutation fixes alone, and the second and third mutations fix together, we apply a two-allele model, another two-allele model, and then a three-allele model.
- If the first two mutations fix together and the third fixes alone, we apply a two-allele model, a three-allele model, and then a two-allele model.
- If all three fix together, we apply a two-allele model, a three-allele model, and then a four-allele model.

Denoting the time of the $i^{\text{th}}$ mutation by *t_i_*, the first of the submodels is applied during the interval $\left[t_{1},t_{2}\right)$, the second during the time interval $\left[t_{2},t_{3}\right)$, and the third during the time interval $\left[t_{3},t_{b}\right]$.

Probabilities are calculated for each possible history, which involves the exponentiation of the three generator matrices of the *k*-allele submodels, which have dimensions $\left(\begin{array}{c} N+k\\ k \end{array}\right)\times \left(\begin{array}{c} N+k\\ k \end{array}\right)$. Each of the *k*-allele submodels reflects the possibility that the population is evolving with *k* different possible states for the *N* individuals at a particular point in time. For general *K* substitutions, we consider $2^{K-1}$ histories. Each of the histories represents a possible path of substitutions that could have occurred in the population and is consistent with the substitutions observed at the end of the branch. A history can be denoted by a *K—*1 digit binary number, with the $i^{\text{th}}$ being equal to 1 if a fixation occurs between the $i^{\text{th}}$ and ${\left(i+1\right)}^{\text{th}}$ mutations—a final fixation between the $K^{\text{th}}$ mutation and the end of the branch is implied. Each history is associated with *K* submodels, the $i^{\text{th}}$ of which models the process between the $i^{\text{th}}$ and ${\left(i+1\right)}^{\text{th}}$ mutations.

### k-Allele Process

Consider a population of fixed size *N* in which mutations occurs at Poisson rate *u_m_* in each individual. New mutations arise in the population at the overall rate *Nu_m_*. When a new mutation occurs, it is assumed that it occurs in a different site to any previous mutation, and hence leads to a never before seen haplotype (as in the infinite sites model (Kimura 1969)). When such a population has *k* unique alleles, we model its evolution until the time of the next mutation using what we here call the *k*-allele process.

We denote the fitness of the $i^{\text{th}}$*mutation*, relative to the wild-type allele at its locus as *f_i_*. The fitness of the $i^{\text{th}}$*haplotype*, denoted $f_{i}^{*}$ is the product of the fitness of the $1^{\text{st}},2^{\text{nd}},\ldots ,i^{\text{th}}$ mutations, which are all present in the $i^{\text{th}}$ haplotype by the assumptions of our model. That is,

(4) $$f_{i}^{*}=\prod_{j=1}^{i}f_{j}.$$

We introduce the vector notation $\underline{f}={\left[f_{i}\right]}_{i=1,\ldots ,k}$ and ${\underline{f}}^{*}={\left[f_{i}^{*}\right]}_{1,\ldots ,k}$ to track the mutation and haplotype fitnesses, respectively.

The *k*-allele process is a *k*-allele continuous-time Moran model. That is, a continuous-time Markov chain $\left\{X_{k}(t;f_{1},f_{2},\ldots ,f_{k-1}))\right\}$ with state space $S_{k}=\{(x_{1},\ldots ,x_{k-1}):x_{j}\in N^{+},\sum_{j}x_{j}\leq N\}$, and generator matrix $Q({\underline{f}}^{*})=[q_{\underline{x}\underline{y}}({\underline{f}}^{*})]$, where the nonzero off-diagonals are given by

(5) $$q_{\underline{x}\underline{y}}({\underline{f}}^{*})=\{\begin{array}{cc} \frac{N-\sum_{j}x_{j}}{N-\sum_{j}x_{j}+\sum_{j}x_{j}f_{j}^{*}}\frac{x_{l}}{N} & \;\text{if}\;\underline{y}=\underline{x}-{\underline{e}}_{l}\\ \frac{x_{l}f_{l}^{*}}{N-\sum_{j}x_{j}+\sum_{j}x_{j}f_{j}^{*}}\frac{N-\sum_{j}x_{j}}{N} & \;\text{if}\;\underline{y}=\underline{x}+{\underline{e}}_{l}\\ \frac{x_{l}f_{l}^{*}}{N-\sum_{j}x_{j}+\sum_{j}x_{j}f_{j}^{*}}\frac{x_{u}}{N} & \;\text{if}\;\underline{y}=\underline{x}+{\underline{e}}_{l}-{\underline{e}}_{u}, \end{array}$$

where ${\underline{e}}_{i}$ represents a vector of zeroes with a 1 in the $i^{\text{th}}$ position. The state of the *k*-allele process is a count of the individuals with each of the *k* possible alleles at any time.

The first case in equation (5) represents the death of an individual carrying the wild-type haplotype and the birth of an individual carrying the $l^{\text{th}}$ haplotype, and the second case is the reverse situation. The third case represents the death of an individual carrying the $u^{\text{th}}$ haplotype and the birth of an individual carrying the $l^{\text{th}}$.

Within each case, the factor on the left accounts for birth with fecundity selection. The probability that an individual carrying the $j^{\text{th}}$ haplotype is selected to reproduce is given by the total fitness contribution of haplotype *j* divided by the total fitness of the population. The factor on the right is a term for death, with any individual in the population being equally likely to die as any other.

Time is scaled such that we expect 1 birth/death event per unit time, including the birth and death of individuals of the same type (which does not lead to any transition of the process). This leads to *N* time units being roughly equivalent to 1 generation in a discrete-time population model. Typically in a phylogenetic analysis, time is measured in substitutions per site, which can vary depending on various factors, including the size of the population and selective effects. For neutral mutations that arise in proportion to the population size, the rate of substitution is independent of *N*. A given mutation, after being introduced, will have a fixation rate dependence on the size of the population. Here an individual in the population is expected to be replaced for each unit of time, and hence *N* time units corresponds roughly to a single generation. Readers should keep this difference in time measurements in mind as they consider the methodology.

### Case with *K *=* *2 Substitutions on a Branch of Length *t_b_*

Now consider the situation in which we know a priori that exactly *K *=* *2 substitutions have occurred along a branch of length *t_b_*. We are interested in evaluating the relative probability that these two mutations will have fixed together, as opposed to one mutation having fixed before the other occurred. We model the process of substitution occurring over this branch by applying several different *k*-allele processes at different points along the branch. Previously, Tataru et al. (2017) considered a biallelic polymorphism aware Wright–Fisher model for the examination of loci under selection. Here, we present an alternative approach with different assumptions. We ignore any mutations which did not go to fixation on the branch, which is to say we explicitly assume no such mutations occurred. It should be noted that such “ghost” mutations can in principle be accounted for as a background distribution of segregating changes without explicit specification. This could be achieved by treating the fitness of the ancestral (wild type) allele as a frequency weighted average of alleles which were segregating during the period over which the substitutions were introduced. Such a calculation to estimate this was recently presented by Galeota-Sprung et al. (2020), which shows the distribution of fitnesses of alleles that are segregating and destined to be lost from the population. In principle, these “ghost” mutations could be accounted for by applying the average fitness calculated in Galeota-Sprung et al. (2020) in place of the normalized “wild-type” fitness applied here. The overall effect of this would be to increase the relative fitness of any particular mutation, increasing the chance of substitution. Accounting for this effect would be important in applications to derive real fitnesses from empirical data; however, for the purposes of this analysis, we keep things simple by assuming that new mutations segregate against a population with fitness normalized to 1.

We start by considering the case in which the arrival times of the mutations are fixed, which we will relax later on. Throughout the following argument, all probabilities are conditioned on these arrival times. Suppose that the first mutation arrived at a time *t*_1_, and that the second arrived at time *t*_2_. We can model the situation from time *t* = *t*_1_ to time *t* = *t*_2_ with the two-allele process. The initial distribution in this interval has all of its mass in the State 1, since a single mutation has just occurred. After time *t* = *t*_2_, there are two cases that need to be considered:

Case 1: the first mutation fixed by time *t*_2_, and then the second mutation fixed by time *t_b_*.

Case 2: the first mutation did not fix (or go extinct) by time *t*_1_, but then the haplotype carrying both the first and second mutation fixed before *t_b_*.

Considering the first of the two cases above, the probability that the two-allele process reached fixation by time *t* = *t*_2_ is given by

(6) $$\begin{array}{c} p_{I}=P(\text{first}\;\text{mutation}\;\text{fixes}\;\text{by}\;t_{2})=P(X_{2}(t_{2}-t_{1};f_{1})=N|X_{2}(t_{1};f_{1})=1)\\ ={\left[{\underline{e}}_{1}e^{Q(f_{1})(t_{2}-t_{1})}\right]}_{N}, \end{array}$$

and when this fixation event occurs the new “wild-type” has fitness *f*_1_. We can then model the population from *t*_2_ to *t_b_* with the two-allele process $X_{2}(\cdot ;f_{2})$. Thus, the probability that the first and second mutations fix independently is given by

(7) $$\begin{array}{c} P(\text{fix}\;\text{independently})=P(X_{2}(t_{2}-t_{1};f_{1})=N,X_{2}(t_{b}-t_{2};f_{2})=N|X_{2}(t_{2};f_{1})=1)\\ =P(X_{2}(t_{2}-t_{1};f_{1})=N|X_{2}(t_{2};f_{1})=1)\times P(X_{2}(t_{b}-t_{2};f_{2})=N|X_{2}(t_{2}-t_{1};f_{1})=N,X_{2}(t_{2};f_{1})=1)\\ ={\left[{\underline{e}}_{1}e^{Q(f_{1})(t_{2}-t_{1})}\right]}_{N}\times P(X_{2}(t_{b}-t_{2};f_{2})=N|X_{2}(t_{2};f_{2})=1)\\ ={\left[{\underline{e}}_{1}e^{Q(f_{1})(t_{2}-t_{1})}\right]}_{N}\times {\left[{\underline{e}}_{1}e^{Q(f_{2})(t_{b}-t_{2})}\right]}_{N} \end{array}$$

Now consider the second case, the probability that there are i=1,…,N−1 individuals carrying the first mutation at time *t*_2_ is given by

(8) $$P(X_{2}(t_{2}-t_{1};f_{1})=i|X_{2}(t_{1};f_{1})=1)={\left[{\underline{e}}_{1}e^{Q(f_{1})(t_{2}-t_{1})}\right]}_{i}.$$

Further, the probability that the second mutation occurs in an individual carrying the first mutation, which we will call occurring in the same lineage and denote by *E_L_*, given that there are *i* such individuals is given by

(9) $$P(E_{L}|i)=\frac{i}{N}.$$

In such event, the population can be modeled during the time from *t*_2_ to *t_b_* by the three-allele process $X_{3}(\cdot ;f_{1},f_{2})$, started from the state [i−1,1], since after the second mutation there will be *i−* 1 individuals carrying only the first mutation and 1 carrying both. The probability that there were *i* individuals carrying the first mutation at time *t*_2_, and that the haplotype carrying both mutations then went to fixation by time *t_b_* is given by

(10) $$\begin{array}{c} P(X_{2}(t_{2}-t_{1};f_{1})=i,X_{3}(t_{b}-t_{2})=[0,N],E_{L}|X_{2}(t_{1};f_{1})=1)\\ =P(X_{2}(t_{2}-t_{1};f_{1})=i,E_{L}|X_{2}(t_{1};f_{1})=1)\times P(X_{3}(t_{b}-t_{2})=[0,N]|X_{2}(t_{1};f_{1})=1,E_{L},X_{2}(t_{2}-t_{1};f_{1})=i)\\ =P(X_{2}(t_{2}-t_{1};f_{1})=i|X_{2}(t_{1};f_{1})=1)\times P(E_{L}|X_{2}(t_{2}-t_{1};f_{1})=i,X_{2}(t_{1};f_{1})=1)\\ \times P(X_{3}(t_{b}-t_{2})=[0,N]|X_{2}(t_{1};f_{1})=1,E_{L},X_{2}(t_{2}-t_{1};f_{1})=i)\\ =P(X_{2}(t_{2}-t_{1};f_{1})=i|X_{2}(t_{1};f_{1})=1)\times P(E_{L}|i)\times P(X_{3}(t_{b}-t_{2})=[0,N]|X_{3}(t_{2})=[i-1,1])\\ ={\left[{\underline{e}}_{1}e^{Q(f_{1})(t_{2}-t_{1})}\right]}_{i}\times \frac{i}{N}\times {\left[{\underline{e}}_{\left[i-1,1\right]}e^{Q(f_{1},f_{2})(t_{b}-t_{2})}\right]}_{\left[0,N\right]}. \end{array}$$

Summing over i=1,…,N−1, we see that the probability that the two mutations fix together is given by

(11) $$p_{T}=P(\text{fix}\;\text{together})=\sum_{i=1}^{N-1}\left({\left[{\underline{e}}_{1}e^{Q(f_{1})(t_{2}-t_{1})}\right]}_{i}\times \frac{i}{N}\times {\left[{\underline{e}}_{\left[i-1,1\right]}e^{Q(f_{1},f_{2})(t_{b}-t_{2})}\right]}_{\left[0,N\right]}\right).$$

The probability that the two mutations fix together, conditional on the two mutations fixing is given by

(12) $$\begin{array}{c} P(\text{fix}\;\text{together}|\text{both}\;\text{fix})=\frac{P(\text{fix}\;\text{together},\text{both}\;\text{fix})}{P(\text{both}\;\text{fix})}\\ =\frac{P(\text{fix}\;\text{together})}{P(\text{fix}\;\text{together})+P(\text{fix}\;\text{independently})}\\ =\frac{p_{T}}{p_{T}+p_{I}}. \end{array}$$

Notice that if we were to assume independent sites, we would calculate the probability of fixation of the two mutations as *p_I_*. If we think of this assumption as an approximation, and the linked-sites model as the “true” model, then equation (12) can be interpreted as the relative error introduced by the approximation. The absolute error is given by *p_T_*. Thinking in these terms, the probability of fixation is always underestimated by the independent sites model on branches with two substitutions, and the same reasoning extends to multiple substitutions. It should be noted however that in practical applications it is the long-run probability of independent fixation that is usually applied, which does not necessarily underestimate the probability of fixing together or separately before the end of the branch. The weak mutation assumption gives each mutation an infinite time in which to fix or go extinct before the next mutation, which itself leads to an overestimation of *p_I_*. Ideally, the overestimation of *p_I_* would balance out the underestimation of the probability of fixation, but this would only very rarely be the case by coincidence.

Since it is not generally known (or inferred) at what time particular mutations were introduced along a branch, we also calculate the relative probability of mutations fixing together versus fixing separately without conditioning on the arrival times (or order) of the mutations. Writing the event that the two mutations fix as *E_F_*, and the event that they fix together as *E_T_*, we can rewrite equation (12) making the conditioning on particular times at which mutations occur explicit,

(13) $$P(E_{T}|t_{1},t_{2},E_{F})=\frac{p_{T}}{p_{T}+p_{I}}.$$

Now, applying the law of total probability we have

(14) $$\begin{array}{c} P(E_{T}|E_{F})=\int_{0}^{t_{b}}\int_{0}^{t_{b}}P(E_{T}|t_{1},t_{2},E_{F})P(t_{1},t_{2})dt_{1}dt_{2}\\ =\int_{0}^{t_{b}}\int_{t_{1}}^{t_{b}}P(E_{T}|t_{1},t_{2},E_{F})P(t_{1},t_{2})dt_{2}dt_{1}\\ +\int_{0}^{t_{b}}\int_{t_{2}}^{t_{b}}P(E_{T}|t_{2},t_{1},E_{F})P(t_{2},t_{1})dt_{1}dt_{2}. \end{array}$$

Notice that in the last line of equation (14), we have split the integral into the two alternatives where the “first” mutation arrives first, and the “second” mutation arrives first—this is in keeping with the preceding results in which we assumed the order of mutations. Note that these two terms are essentially the same, but with the roles of *t*_1_ and *t*_2_, as well as *f*_1_ and *f*_2_ reversed (as indicated by our reordering of the arguments).

Since $P(E_{T}|t_{1},t_{2},E_{F})$ is given by equation (13) we need only find $P(t_{1},t_{2})$ in order to have an expression for the integrand (which we will proceed to integrate numerically). Since we assume that mutations arrive at (the same) Poisson rate, a well-known result (e.g., Ross 1996, p. 66) gives that the joint distribution of arrival times is

(15) $$P(t_{1},t_{2})=\frac{1}{t_{b}^{2}},$$

where the usual factor accounting for the order of the random variables is dropped, since we have fixed the order within the two terms of equation (14).

### Computing for Large *N*

The calculation of $P(E_{T}|E_{F})$ involves some expensive computations, most notably the integration of terms involving the exponential of the three-allele process’s generator matrix. Attempting these computations without special consideration is infeasible for realistic values of *N*. At the time of writing, we have not found a suitable approximation or computational approach to allow us to obtain $P(E_{T}|E_{F})$ for large *N*. Nonetheless, we detail some particularly efficient approaches to compute fixation probabilities in the two-allele process, and a relatively efficient procedure to compute fixation probabilities in the general three-allele process for long branches.

The two-allele Moran model is one of the simplest models in population genetics from a computational perspective and has received a lot of attention since its introduction by Moran in 1958 (Moran 1958). Often diffusion approximations are used for the Moran model and closely related Wright–Fisher model, most notably the results derived by Kimura (1969). Here we take a slightly different approach to compute the exact long-run fixation probability using standard methods from the theory of continuous-time Markov chains, as well as applying a recent approximation due to Hathcock and Strogatz (2019) for the finite-time case.

The case where the first mutation is segregating for a long time before the second mutation arises can be approximated by the long-run fixation probability as $t_{2}\to \infty$. The exact long-run fixation probability is given by

(16) $$p_{\text{fix}}^{\infty }=\underset{t\to \infty }{\lim}P(X_{2}(t)=N|X_{2}(0)=1)=-{\underline{e}}_{1}{\left(Q^{*}\right)}^{-1}{\underline{v}}_{N},$$

where,

(17) $$Q=\left[\begin{array}{cc} Q^{*} & V\\ O & O \end{array}\right],$$

and $Q^{*}$ contains the transition rates between the transient states while V contains the transition rates into the absorbing states. The matrix $-{\left(Q^{*}\right)}^{-1}$ records the expected times spent in each transient state before absorption Darroch and Seneta (1967), while ${\underline{v}}_{N}$ records the rate at which absorption into state *N* occurs from each transient state.

This can be solved very efficiently using standard linear algebra techniques. Specifically, we can rewrite equation (16) as

(18) $$p_{\text{fix}}^{\infty }=-{\left(Q^{*}\right)}_{1}^{-1}{\underline{v}}_{N},$$

where ${\left(Q^{*}\right)}_{1}^{-1}$ denotes the first row of the inverse of $Q^{*}$. Thus, we need only solve the system of linear equations

(19) $${\left(Q^{*}\right)}_{1}^{-1}Q^{*}={\underline{e}}_{1},$$

which is considerably less computationally expensive than computing the inverse $-{\left(Q^{*}\right)}^{-1}$ itself.

We note that this approach to calculating $p_{\text{fix}}^{\infty }$ is roughly equivalent to the method applied to the Wright–Fisher model in Krukov et al.’s Wright–Fisher Exact Solver (WFES) (Krukov et al. 2017), but applied in our case to the continuous-time process. Other results equivalent to those calculated by WFES (Krukov et al. 2017) can be obtained for continuous-time processes in a straightforward manner (refer to the supplement of that paper and De Sanctis et al. (2017)). In the case of the Moran model, exact computation is more efficient than it is for the Wright–Fisher model (as noted by De Sanctis et al. (2017)) since the generator matrix $Q^{*}$ is tridiagonal, leading to effectively instantaneous computations on our workstation computer for *N* up to about 10^6^. Alternatively, the fixation formula derived for the diffusion approximation by Kimura (1969) can be used in place of $p_{\text{fix}}^{\infty }$, which saves time when *N* is much larger than 10^6^, but becomes inaccurate when the effect of selection is large.

If the first mutation segregates for a short enough time that the probability of fixation before the arrival of the second mutation is not well approximated by the long-run fixation probability, then we apply the approximation due to Hathcock and Strogatz (2019). In the present work, we have a slightly different implementation of the Moran model (namely, our model is in continuous-time and allows for an individual to both give birth and die at any instant). This leads to some differences in the argument, but the same result is obtained in the two-allele case.

Hathcock and Strogatz (2019) showed that as N→∞

(20) $$\frac{T_{F}-\mu }{\sigma }\overset{d}{\to }\frac{G_{1}+fG_{2}}{\sqrt{1+f^{2}}},$$

where *T_F_* is a random variable denoting the time of fixation, conditional on eventual fixation, *μ and σ* are it’s expectation and variance, respectively, *G*_1_ and *G*_2_ are two independent Gumbel distributed random variables, and *f* is the fitness of the proposed mutation relative to that of the wild type.

Thus, letting

(21) $$G=\frac{G_{1}+fG_{2}}{\sqrt{1+f^{2}}}$$

We can approximate the cumulative distribution function of *T_F_*, $F_{T_{F}}(t)$ by

(22) $$F_{T_{F}}(t)\approx F_{G}\left(\frac{t-\mu }{\sigma }\right).$$

Hence, we can approximate the probability that a mutation has gone to fixation by time *t* using

(23) $$\begin{array}{c} p_{\text{fix}}(t)=P(T_{F}<t|X(0)=1)\\ =P(T_{F}<t|X(0)=1,T_{F}<\infty )P(T_{F}<\infty )\\ =F_{T_{F}}(t)p_{\text{fix}}^{\infty }\\ \approx F_{G\prime }\left(\frac{t-\mu }{\sigma }\right)p_{\text{fix}}^{\infty }. \end{array}$$

We can obtain *μ and σ* quite efficiently, since (Aalto 1989)

(24) $$\mu =-\sum_{j}[{\left(Q^{*}\right)}_{1}^{-1}{]}_{j}\frac{E(T|X(0)=j)}{E(T|X(0)=1)},$$

and $E(T|X_{0}=i)$, the mean time to absorption given *i* initial mutants can be calculated using

(25) $$E(T|X_{0}=i)={\left[-{\left(Q^{*}\right)}^{-1}V\right]}_{i}.$$

A similar approach can be applied to the variance, with

(26) $$\sigma^{2}=2\sum_{j}[{\left(Q^{*}\right)}_{1}^{-2}{]}_{j}\frac{E(T|X(0)=j)}{E(T|X(0)=1)}-\mu^{2},$$

where

(27) $${\left(Q^{*}\right)}_{1}^{-2}Q^{*}={\left(Q^{*}\right)}_{1}^{-1}.$$

Calculating the mean and variance from the above equations using the MATLAB operator “\”, takes under a second for *N* on the order of 10^6^ on our i7-8700 desktop computer, but takes several seconds when *N* is on the order of 10^7^. We then calculate *F_G_* numerically, which is effectively instantaneous with no parameter dependence.

**Fig. 1.**
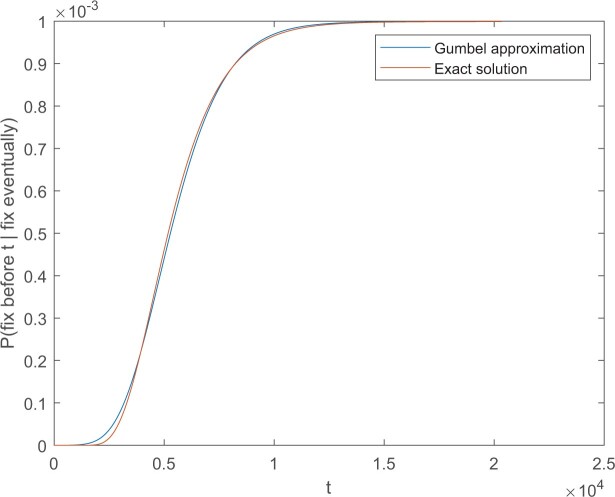
Gumbel approximation versus exact solution for cumulative distribution of time to fixation given eventual fixation in the two-allele process with $N=10^{4}$, *Ns* = 10. Units of time are expected time to replace one member of the population.

Hathcock and Strogatz’s analysis (Hathcock and Strogatz 2019) shows that the approximation in equation (22) is highly accurate for large selective coefficients (*Ns* = 500) even with *N* as small as 5000. Investigating some cases, we find that even with *N *=* *100 the exact and approximate curves in equation (23) are indistinguishable by eye. The fit remains quite good with *Ns* = 10 and $N=10^{4}$, as shown in figure 1. It is reasonable to think that for realistically large values of *N*, this approximation is more than sufficient for any practical purpose.

When all that is required is the distribution of time-to-fixation for the two-allele process, the above procedure is an efficient way to obtain it. However, in order to apply a similar approximation for the three-allele process, the full distribution at the time of the second mutation is required. In this case, Expokit (Sidje 1998) provides an efficient means to compute the full distribution. Expokit is a software package which allows the solution of matrix exponential equations involving very large matrices using Krylov approximation. In particular, the function “mexpv” solves for the full distribution of a CTMC at some time *t* in the future, given an initial distribution and the generator matrix Q.

The more significant computational difficulties appear when *k *>* *2. We can use the same approach to the two-allele model above to compute the probability of fixation in the limit as $t_{b}\to \infty$, and this provides a suitable approximation when *t_b_* is large. Given the initial distribution is $\underline{\alpha }$, we can calculate the exact long-run fixation probability by

(28) $$p_{\text{fix}}^{\infty }=-{\left(Q^{*}\right)}_{\cdot }^{-1}{\underline{v}}_{\left(0,\ldots ,N\right)},$$

where ${\left(Q^{*}\right)}_{\cdot }^{-1}$ is computed by solving

(29) $${\left(Q^{*}\right)}_{\cdot }^{-1}Q^{*}=\underline{\alpha }.$$

However, the size of the system of equations grows combinatorially with *k*, and so is not suitable for *k* much larger than 3.

In order to make further progress in computing for large *N*, an approximation for the probability of fixation in the *k*-allele process over short times is required. A promising approach is to replace the *k*-allele processes discussed here with appropriate diffusion approximations, which could in principle allow for the application of models accounting for the effects of linked sites for the estimation of amino acid fitness profiles.

### Case with *K *>* *2 Substitutions on a Branch of Length *t_b_*

We now consider the case where substitutions occur at *K* sites for arbitrary fixed *K*. Again, we consider only those mutations that fix. We represent the history of fixation events along a branch with *K* substitutions by a (K−1)-digit binary number whose $i^{\text{th}}$ digit is a 1 if a fixation event occurs between the $i^{\text{th}}$ and ${\left(i+1\right)}^{\text{th}}$ mutation events. The final fixation event (between the $K^{\text{th}}$ mutation and the end of the branch) is implied. For example, with *K *=* *4 one possible history is *H *=* *001, under which a fixation occurs between the third and fourth mutations, so that the first three mutations fix together, and then the fourth mutation fixes alone. Clearly, there are $2^{K-1}$ such possible histories.

We write the $i^{\text{th}}$ digit of *H* as *H_i_*, and the first *i* digits of *H* as $H^{\left(i\right)}$, which we call the $i^{\text{th}}$ subhistory of *H*. For example, for *H *=* *101, $H_{2}=0$, and $H^{\left(2\right)}=10$. For a branch with fixed *K*, and fixed mutation times *t_i_*, we wish to evaluate the probability of each possible history for the branch.

We track only the component of the transient distribution associated with a particular history *H* at any time *t*, which is given in terms of the *k*-allele process that is applied at this time for this history. The relevant model for $t\in [t_{i},t_{i+1})$ is a (2+i−κ)-allele process where $\kappa =\max\{1,j\leq i:H_{j-1}=1\}$, with parameters $f_{\kappa },f_{\kappa }f_{\kappa +1},\ldots ,\prod_{j=\kappa }^{i}f_{j}$.

Since we only evaluate histories which lead to fixation of all *K* mutations, any mutation which is no longer being tracked must have already fixed. Thus, we can compute the probability of all possible histories that lead to eventual fixation of all *K* mutations, and the sum of these probabilities is the probability of fixation of all *K* mutations (since the histories form a partition of the event that all *K* mutations fix). Note, this is a generalization of the procedure for the case *K *=* *2, where the two possible histories are “fix together” and “fix separately.”

The distribution for $t\in [t_{1},t_{2})$, for any history *H* is given in terms of the two-allele process by

(30) $${\underline{\alpha }}^{H}(t;\underline{t})={\underline{e}}_{1}e^{Q(f_{1})(t-t_{1})},$$

where $\underline{t}=[t_{1},t_{2},\ldots ,t_{K},t_{b}]$.

The distribution for $t\in [t_{i},t_{i+1})$ for i=2,…,K−1, or $t\in [t_{K},t_{b}]$ is split into components associated with each ${\left(i-1\right)}^{\text{th}}$ subhistory.

If $H_{i-1}=0$, then the initial distribution of the *k*-allele process applied in the interval $\left[t_{i},t_{i+1}\right)$ is given by,

(31) $${\left[{\underline{\alpha }}^{H}(t_{i};\underline{t})\right]}_{\underline{x}}=\sum_{\left\{\underline{y},j:(\underline{y}-{\underline{e}}_{j})⌢[1]=\underline{x}\right\}}\frac{y_{j}}{N}\underset{t\to t_{i}^{-}}{\lim}{\left[{\underline{\alpha }}^{H}(t;\underline{t})\right]}_{\underline{y}},$$

where ⌢ denotes vector concatenation. Note that for fixed $\underline{x}$ each $\underline{y}$ is associated with at most one *j* in the sum above. The summand is the probability that the process is in state $\underline{y}$ (in terms of a *k*-allele process) multiplied by the probability that a mutation introduced when the process is in state $\underline{y}$ leads to state $\underline{x}$ (in terms of a (k+1)-allele process).

If $H_{i-1}=1$, then the initial distribution of the two-allele process applied in the interval $\left[t_{i},t_{i+1}\right)$ is given (element-wise) by

(32) $${\underline{\alpha }}^{\left(H\right)}(t_{i};\underline{t})=\underset{t\to t_{i}^{-}}{\lim}{\left[{\underline{\alpha }}^{H}(t;\underline{t})\right]}_{\left(0,0,\ldots ,N\right)}{\underline{e}}_{1}.$$

In either case, the distribution for the rest of the interval—that is, for $t\in (t_{i},t_{i+1})$ (or $t\in (t_{K},t_{b}]$ for the last interval) is given by

(33) $${\underline{\alpha }}^{H}(t;\underline{t})={\underline{\alpha }}^{H}(t_{i};\underline{t})e^{Q(f_{\kappa },f_{\kappa +1},\ldots ,f_{i})(t-t_{i})},$$

where $\kappa =\max\{1,j\leq i:H_{j-1}=1\}$.

Finally, the probability of a history *H* is given by

(34) $$P(H|t_{1},..,t_{K},t_{b})={\left[{\underline{\alpha }}^{H}(t_{b};\underline{t})\right]}_{\left(0,0,\ldots ,N\right)},$$

and summing this expression over all *H* gives the probability of fixation of all *K* mutations. Notice that assuming independent sites, only one of these histories is accounted for in the calculating of the probability of fixation of all *K* mutations. Again, the caveat being that in practice each mutation is given an infinitely long period during which to fix.

We can extend this to random mutation times in a manner analogous to the case where *K *=* *2. We no longer treat the order in which mutations arise as fixed—the vector of mutation times $\underline{t}={\left[t_{i}\right]}_{i=1,..,K}$, is such that *t_i_* corresponds to the time of the mutation with fitness *f_i_*. Assuming that mutations arise at a Poisson rate, then conditional on *K* arrivals on a branch of length *t_b_*, mutation times are uniformly distributed. It follows that the probability of a history *H* is given by.

(35) $$\begin{array}{c} P(H|t_{b})=\frac{1}{t_{b}^{K}}\int_{T}P(H|\underline{t})d\underline{t}\\ =\frac{1}{t_{b}^{K}}\int_{T}[{\underline{\alpha }}^{H}(t_{b};\underline{t}){]}_{\left(0,0,\ldots ,N\right)}d\underline{t} \end{array},$$

where T denotes the set of vectors $\underline{t}$ with elements in $\left[0,t_{b}\right]$.

Notice that for two histories *H* and H′ which share the same $i^{\text{th}}$ subhistory we have

(36) $${\underline{\alpha }}^{H}(t;\underline{t})={\underline{\alpha }}^{H\prime }(t;\underline{t}),\;\text{for}\;\text{all}\;t<t_{i},$$

which can be applied to reduce the computational requirements of evaluating probabilities for the full set of histories.

## Discussion

In this article, we have presented an analysis of the probability that substitutions introduced in some population (or on some branch of a phylogenetic tree) would have gone to fixation simultaneously as opposed to fixing sequentially. We make some strong assumptions about the evolution of the population over the branch, most notably we ignore any mutations segregating in the background besides those which go to fixation. Recent work by Galeota-Sprung et al. (2020) has derived the distribution of expected fitness effects caused by background mutations across the genome. In principle, the effect of background mutations could be accounted for by assuming that the introduced substitutions segregate against individuals with the average fitness derived by Galeota-Sprung et al. (2020). In effect, this would act to increase the relative fitness of any given mutation, increasing the probability of fixation. For the purposes of the analysis presented here, we do not feel that it is necessary to account for this effect. However, in the case where results are applied to infer real fitness effects, it would be worthwhile to account for this effect either using the full distribution derived by Galeota-Sprung et al. (2020) or applying the average as the fixed fitness against which new mutations segregate as discussed here.

Another notable assumption that we make in the infinite sites model is that the population at time 0 (i.e., the beginning of a branch) is homogeneous, and similarly we analyze only the probability of “fixation” in the sense that the population is again homogeneous at the end of the branch. In reality, there are likely to be multiple alleles segregating at the beginning and the end of the branch. In our approach, the focal sites are those that go to fixation and the equilibrium distribution of other segregating alleles could be partly accounted for by the application of the results of Galeota-Sprung et al. (2020) as discussed above. The finite sites model that was also presented, while suffering from other limitations, does not make the assumptions about evolution over the branch that need this type of correction.

For the case of a branch upon which two substitutions occur, we have computed the relative probability that the two substitutions fix together versus fix separately for various different fitness values with both fixed and random mutation times for various branch lengths under our infinite sites substitution model with complete linkage.

**Fig. 2.**
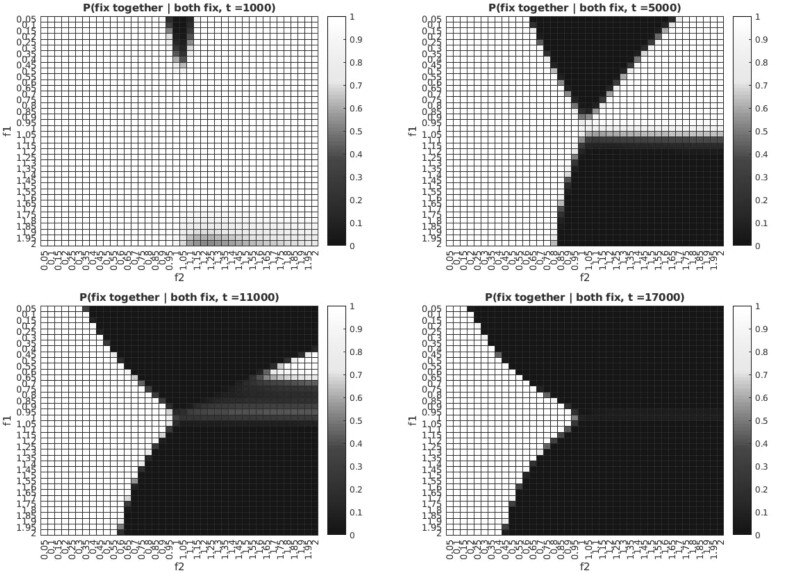
Probability that two mutations fix together conditional on both reaching fixation eventually for various fixed time-between-mutations *t* (units of average time to replace an individual in the population) as a function of *f*_1_ (fitness of first mutation to arise relative to wild-type) and *f*_2_ (fitness of second mutation to arise relative to wild-type) with *N *=* *100.

For fixed mutation times, we observe that, conditional on fixation, the probability that the two mutations go to fixation together is near 1 or 0 for most of the parameter space, with a sharp boundary between the two modes which depends on the time-between-mutations. These regions correspond respectively to a region in which the independent-sites model is highly mis-specified, and correctly specified. Two mutations which fix separately can be modeled as independent, while two that fix together cannot. For small time-between-mutations, the region of the parameter space in which the independent-sites model is mis-specified is largest, with regions in which the assumption of independence is valid appearing and growing as the time-between-mutations becomes larger. Figure 2 shows the long-run conditional probability of fixing together given fixation as a function of *f*_1_, *f*_2_ for four times-between-mutations *t*.

**Fig. 3.**
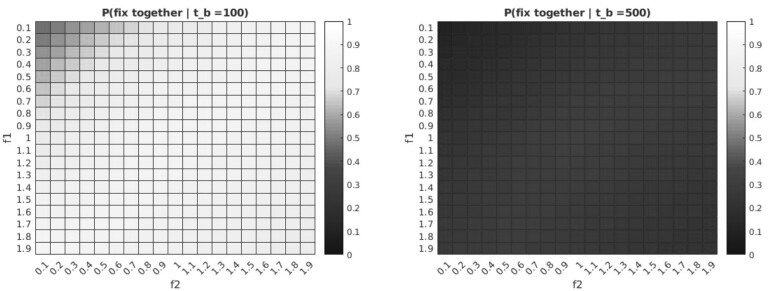
Probability that two mutations (arising according to a Poisson process) fix together conditional on exactly two mutations arising, and both reaching fixation eventually on a branch of length *t_b_* (units of average time to replace an individual in the population) as a function of their (unordered) fitnesses relative to the wild-type *f*_1_, *f*_2_ with *N *=* *10.

For (random) variable mutation times, we observe that conditional on fixation, the probability that the two mutations go to fixation together is largely dependent on the branch length *t_b_* (which in biological terms corresponds to the substitution rate). While branch lengths measured in amino acid distance would make this inference impossible, branch lengths measured in synonymous site substitution rates would enable independent estimation of the branch length and the number of amino acid substitutions. For short branch lengths, the two mutations are most likely to fix together, and for longer branch lengths they are most likely to fix separately. For a given *t_b_*, the highest conditional probability of fixing together occurs where both mutations are associated with neutral fitnesses, while the lowest probability is associated with mutations which are both associated with fitnesses less than one. Figure 3 shows these results for three different branch lengths. Note that here we do not assume an order for the arrival of mutations, hence the symmetry about the line *f*_1_ = *f*_2_.

These results are consistent with the observation that selectively neutral alleles of a given frequency tend to have been segregating for longer than alleles under either positive directional or negative selection (Platt et al. 2019). The observation that fixing together is most likely for neutral alleles could be explained by such alleles segregating for longer, providing a longer window before fixation for the second mutation to arise. It should be noted that these neutral changes are the substitutions where the selective effects are least expected to be mis-estimated.

Notably, the conditional probability of the two mutations fixing together is not symmetric about the line $f_{1}+f_{2}=2$, as might be expected from (but is not implied by) the results in (Maruyama 1974). The intuition for expecting symmetry is that the age distribution of alleles observed at a given frequency under selection is independent of the direction of selection. As such the window of opportunity for a second mutation to arise in the same lineage as the first is identical for $f_{1}=1+\epsilon$ and $f_{1}=1-\epsilon$. However, this line of reasoning neglects the effect of fitness (and multiplicative fitnesses in particular) on the probability of fixation. When both mutations are advantageous the combined allele is relatively more likely to fix than either single-mutation allele would be, while the reverse is true for disadvantageous mutations. Hence, even when conditioning on eventual fixation, we see the asymmetry about $f_{1}+f_{2}=2$.

We also note that the conditional probability of fixing together can be interpreted as the relative error introduced by modeling sites as independent if we treat the linked-sites model as the “true” model. This is applicable for regions of low recombination, where mutations which occur in the same lineage remain linked. We show that in this instance, the independent sites model always underestimates the probability that multiple mutations on the same branch will eventually fix. This would seem to imply consistent overestimation of the fitnesses of amino acids associated with substitutions, regardless of whether a mutation is subject to hitchhiking or background selection. Even if the subsequent mutation is deleterious, the preceding one is nonetheless given an extra opportunity to fix which is not accounted for when assuming that each mutation must fix or become extinct before the next. The caveat is that in practice mutation is usually assumed to be weak, effectively giving each mutation an infinite period over which it can become fixed (or extinct) prior to any subsequent mutation, which leads to overestimation of the probability of independent fixation. While its unlikely in any instance to be the case that these two competing biases cancel, this is the mechanism by which hitchhiking can lead to increased fitness estimates for the beneficiary while background selection can lead to decreased estimates when assuming independent sites.

Given a known substitution history (often called a mutational path (Bollback et al. 2007; Monit and Goldstein 2018)) for each site over a fixed tree, estimating fitness parameters under the model described here is straight-forward in principle. Supposing that we know that substitutions occurred at *K* sites over a particular branch, the likelihood of a set of relative fitnesses $\underline{f}=[f_{1},f_{2},\ldots ,f_{K}]$ (relative fitnesses being the fitness of the substituted character divided by the fitness of the character at the beginning of the branch at that site) is given by

(37) $$L(\underline{f})=\sum_{H\in H}P(H),$$

where *H* is the set of fixation histories which can account for the substitution history. Fixation histories are distinct from substitution histories in the sense that they account for which mutations fix together. It is possible then to use maximum likelihood estimation to estimate fitness parameters which account for the possibility of sites being linked for fixed trees and substitution histories. It is also possible to implement a Bayesian procedure, for example, treating the tree and substitution histories as nuisance parameters and sampling from the distribution of fitnesses, or (*mutatis mutandis*) implementing the model in phylogenetic inference, etc. In practice, this would be highly computationally expensive, and we have not done any such inference here, focusing instead on theoretical results pertaining to the model itself. In order to make such inferences practicable, further consideration of the computational approach, or the application of some approximations to the fixation probabilities of the *k*-allele processes would be necessary.

As models that capture the generative process for amino acid substitution, integrating population genetics, evolutionary biology, and protein biophysics move to maturity, multiple layers of complexity need to be integrated (Teufel et al. 2018). The danger in not doing so is model mis-specification leading to incorrect inference (Liberles et al. 2013). In the context of mutation–selection models, this includes accounting for linkage, accounting for the structural and functional interaction of sites, accounting for the inherently nonequilibrium nature of the evolutionary process (which is necessary for inferring positive directional selection (Spielman and Wilke 2015; Ritchie et al. 2021)), accounting for selection on synonymous sites, and perhaps other processes. The development of these various pieces is an ongoing process and the work here presents the development of the linkage component for this modeling framework. With all of this, we will have more powerful computational inference frameworks for detecting which proteins have changed function between closely related species, a grand challenge problem in comparative genomics.

### Future Directions

The work presented here represents our first steps towards developing a procedure by which amino acid fitness profiles estimated from independent-sites substitution models can be adjusted to account for the effects of genetic linkage.

The results described in this article could in principle be applied to assess the effect of linkage on inferences made from some arbitrary amino acid substitution model which has been fit to a phylogenetic data set. If a reconstructed substitution history has been obtained over the tree, then equation (35) can be evaluated over fixation histories which are compatible with the estimated substitution history. The probability mass associated with fixation histories including linked fixation events then gives an indication of the extent to which linkage effects are present, and hence the extent to which an independent sites substitution model is likely to be misspecified. This in turn can indicate whether selective effects obtained from the substitution model are likely to be underestimated owing to the model not having accounted for the Hill–Robertson effect (Hill and Robertson 1966).

However, there remains a large gap between the theoretical results described here and a practical methodology for re-estimating selective effects to account for linkage. Notably, the results presented in this article are intractable for large populations, and for branches with many substitutions. The first of these problems could be addressed by applying a diffusion model in place of the *k*-allele Moran models discussed above. The other component of this gap lies in the problem of mapping from the probability of linked substitutions developed above to an actual re-estimation of the fitness effects. Although it is widely understood that linkage dampens the effects of selection, the extent of this effect in the context of many substitutions with associated probabilities of linkage needs to be quantified. In principle, updates to fitnesses estimated from an independent sites model could be obtained by estimating fitnesses with a linked sites model on those branches upon which linkage is identified by considering the probability that substitutions fix together. These could then be averaged in some way with the estimates obtained from fitting an independent sites model over the whole tree. Alternatively, a framework could be developed where a linked sites model is fit on appropriate branches, while an independent sites model is fit to the remaining branches, and parameters are estimated over the tree taking both cases into account simultaneously.

Lastly, some nonindependent sites will interact functionally leading to nonadditive interactions. While these can in principle be treated with expanded parameterization associated with standard statistical genetics models for epistasis, a mechanistic opportunity exists to explicitly treat the underlying biophysical processes associated with protein folding and protein interaction to account for selective effects in a distinct modeling trajectory. With all of these pieces, the field is encroaching on biological realism in its treatment of selection in protein evolution as part of the ongoing search for positive directional selection that makes species distinct.
